# Complementary value of transverse plane descriptors in the SRS-Lenke-Aubin 3D classification for adolescent idiopathic scoliosis

**DOI:** 10.1007/s43390-026-01287-0

**Published:** 2026-01-27

**Authors:** Carl-Eric Aubin, Lawrence G. Lenke, Virginie Lafage, Michelle C. Welborn, Justin S. Smith, A. Noelle Larson, Michael G. Vitale, Takashi Kaito, Peter O. Newton, Jeffrey Mullin, Christiane Caouette, Delphine Aubin, Brice Ilharreborde

**Affiliations:** 1 Department of Mechanical Engineering, Polytechnique Montréal, P.O. Box 6079, Downtown Station, Montreal, QC H3C 3A7 Canada; 2 Sainte-Justine University Hospital Center, 3175 Côte Sainte-Catherine Road, Montreal, QC H3T 1C5 Canada; 3 Department of Orthopedic Surgery, Columbia University Vagelos College of Physicians and Surgeons, NewYork-Presbyterian Och Spine Hospital, New York, NY USA; 4 Department of Orthopaedic Surgery, Lenox Hill Hospital, Northwell Health, New York, NY USA; 5 Department of Orthopaedics and Rehabilitation, Oregon Health & Science University School of Medicine, Shriners Children’s Portland, Portland, OR USA; 6 Department of Neurosurgery, University of Virginia School of Medicine, Charlottesville, VA USA; 7 Department of Orthopedic Surgery, Mayo Clinic, 200 First Street SW, Rochester, MN 55905 USA; 8 Department of Orthopedic Surgery, Columbia University Vagelos College of Physicians and Surgeons, NewYork-Presbyterian Morgan Stanley Children’s Hospital, New York, NY USA; 9 Division of Orthopaedic Surgery, Osaka University Graduate School of Medicine, Osaka Rosai Hospital, Osaka, Japan; 10 Department of Orthopedics, Rady Children’s Hospital, 3020 Children’s Way, San Diego, CA 92123 USA; 11 Department of Neurosurgery, Jacobs School of Medicine and Biomedical Sciences, University at Buffalo, The State University of New York, Buffalo, NY USA; 12 AP-HP Hôpital Universitaire Robert-Debré, 48 Boulevard Sérurier, 75019 Paris, France

**Keywords:** Adolescent idiopathic scoliosis, 3D metrics, Transverse plane, Classification, Axial rotation

## Abstract

**Purpose:**

To assess the complementary value of transverse plane descriptors (orientation of the regional planes of deformation (ORPD) and local apical vertebral rotations (AVR)) integrated into the new modular three-tiered, four-modifier SRS-Lenke-Aubin 3D classification, relative to conventional 2D radiographic parameters and current Lenke 2D classification in adolescent idiopathic scoliosis (AIS).

**Methods:**

Transverse plane deformities of 285 surgically treated AIS cases reconstructed in 3D were quantified using ORPD and AVR, independently assessed for the proximal thoracic (PT), main thoracic (MT), and thoracolumbar/lumbar (TL/L) regions. Correlation analyses evaluated relationships between standard 2D parameters (Cobb angles, thoracic kyphosis (TK), lumbar lordosis (LL)) and transverse plane indices (ORPD, AVR). The distribution of ORPD and AVR subclasses was examined, as well as the associations between conventional Lenke lumbar and thoracic sagittal profile modifiers, and their corresponding 3D transverse plane modifiers. Complementary analyses also included 3D displacement of the apex relative to the end-vertebrae line (DAEVL).

**Results:**

Nearly all ORPD–AVR subclass combinations were observed across regions, confirming the system’s ability to capture diverse deformity patterns. ORPD and AVR were independent in PT and MT but correlated in TL/L (r = 0.69). Cobb angle correlated moderately with ORPD in MT (r = 0.43) and strongly in TL/L (r = 0.67), while correlations with AVR were moderate in MT (r = 0.50) and TL/L (r = 0.59). TK correlated negatively with MT ORPD (r = –0.58), whereas LL showed no association with TL/L ORPD. DAEVL correlated strongly with Cobb across all regions but only weakly to moderately with ORPD. Associations between Lenke 2D modifiers and ORPD were strong in TL/L (V = 0.59) and moderate in MT (V = 0.37). Multivariate models showed that Cobb and TK explained ~ 44% of MT ORPD variance, while > 55% of ORPD variability across regions remained unexplained by 2D parameters.

**Conclusions:**

ORPD and AVR provide complementary, region-specific 3D information beyond conventional 2D measures and Lenke modifiers. Their integration into the SRS-Lenke-Aubin 3D classification enhances dimensional completeness while preserving usability, laying the groundwork for future outcome-based evaluations.

## Introduction

Adolescent idiopathic scoliosis (AIS) is a complex three-dimensional (3D) spinal deformity, marked by structural curvatures that span all three anatomical planes—coronal, sagittal, and transverse. While surgical treatment is often warranted in severe or progressive cases, the diagnostic and planning frameworks used in current clinical practice remain predominantly rooted in two-dimensional (2D) radiographic measurements. Conventional assessments emphasize Cobb angles of major and minor curves in the coronal plane and sagittal spinal alignment, but they fail to capture the full extent of transverse plane deformity, including vertebral rotation and torsional imbalance.

Among existing classification systems, the Lenke classification has become the clinical gold standard for surgical planning in AIS. Introduced in 2001, the Lenke system stratifies AIS into curve types (1–6) and incorporates coronal lumbar spine (A, B, C) and thoracic sagittal profile (−, N, +) modifiers to guide posterior spinal fusion strategies [1, 2]. Its structured, reproducible framework has been instrumental in standardizing care across institutions and has contributed significantly to the evolution of AIS surgery [3]. However, the Lenke classification is inherently 2D, lacking explicit consideration of the transverse plane—a domain increasingly recognized as critical to both the biomechanical understanding of AIS and the development of modern corrective techniques—and it does not explore the sagittal alignment of either the lumbar or the proximal thoracic spine.

Advances in imaging technologies and 3D reconstruction methodologies have enabled deeper exploration of the transverse plane [4]. These developments have not only highlighted the clinical relevance of axial vertebral rotation and regional transverse curvature orientation but have also exposed limitations in current classification paradigms. Multiple research efforts have proposed 3D classifications based on vertebral rotation, geometric torsion, or data-driven clustering approaches [5–8], [14], [9–12]. Yet despite their conceptual sophistication, such systems have not been widely adopted in clinical practice due to issues of complexity, limited interpretability, and lack of compatibility with standard imaging workflows [13, 14].

To address this gap, the Scoliosis Research Society (SRS) reactivated its 3D Classification Task Force with a clear mandate: to develop a comprehensible, reproducible, and clinically applicable 3D classification system for AIS that would enhance existing diagnostic frameworks without disrupting established workflows. This initiative culminated in the development of the SRS-Lenke-Aubin 3D classification (Aubin, Lenke et al., 2025), a modular 3-tiered, 4-modifier system extension of the Lenke system that incorporates 2 additional modifiers from the transverse plane: the *orientation of regional planes of deformation* (ORPD) and local *apical vertebral rotation* (AVR). These indices capture both the global orientation of spinal curves and the localized torsional components of the deformity, with separate values assigned for the proximal thoracic (PT), main thoracic (MT), and thoracolumbar/lumbar (TL/L) regions. Designed to complement the original 2D descriptors, the transverse plane modifiers preserve clinical interpretability while offering a more complete 3D representation of spinal architecture.

Preliminary application of this new classification has demonstrated its ability to describe a wide range of AIS presentations, with all combinations of ORPD (1, 2, 3) and AVR (s, m, l) subclasses represented across a surgical cohort (Aubin, Lenke et al., 2025). These findings suggest that the transverse plane descriptors contribute meaningful dimensional information that is not captured by traditional 2D measurements. However, the extent to which these 3D descriptors provide independent and complementary information, relative to conventional radiographic parameters (such as Cobb angles, thoracic kyphosis, and lumbar lordosis) and to the classic Lenke modifiers, remains to be rigorously assessed.

The objective of the present study is to evaluate the independence and complementary value of the newly introduced transverse plane descriptors. Specifically, we aim to investigate whether these indices provide non-redundant and complementary information beyond what is captured by conventional 2D parameters and Lenke classification categories.

## Materials and methods

The SRS-Lenke-Aubin 3D classification and its guiding principles have been comprehensively described and justified in the companion manuscript (Aubin, Lenke et al., 2025), where the full hierarchical three-tiered, four-modifier framework—including its rationale and development process—is presented; the key elements relevant to the present analyses are briefly summarized here. It builds on the original Lenke framework, which includes a primary curve type designation (1–6) and two 3-tiered modifiers: the lumbar modifier (A, B, C), assessing coronal alignment of the lumbar apex, and the thoracic sagittal profile modifier (–, N, +), based on thoracic kyphosis (T5–T12) [1, 2]. The 3D system extends this framework by integrating two transverse plane modifiers for each spinal region (PT, MT, and TL/L), the whole being structured as a 3-tiered, four-modifier framework: curve type + lumbar modifier + thoracic sagittal profile modifier + regional and local transverse plane modifiers (Aubin, Lenke et al., 2025).

**Fig. 1:**
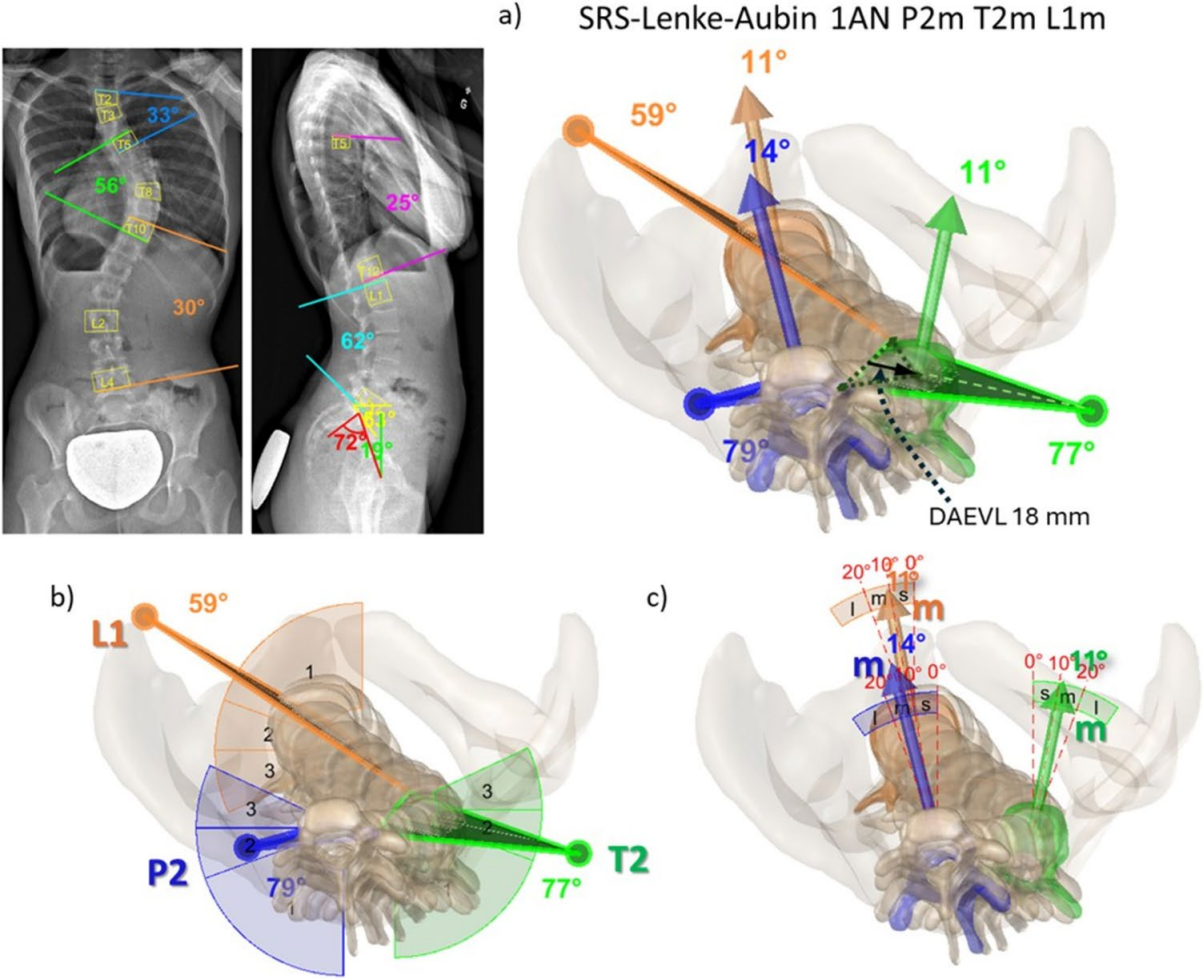
**a** Case example illustrating the integration of the regional (ORPD) and local (AVR) transverse plane indices into the new SRS-Lenke-Aubin 3D Classification. To improve visual clarity and aid in subclass identification, **b** shows a superimposed shaded ruler illustrating ORPD categories, and **c** displays directional AVR arrows, for the PT, MT, and TL/L regions (in blue, green, and orange, respectively)

The transverse plane regional modifier is based on the Orientation of Regional Planes of Deformation (ORPD), defined as the angle between the sagittal plane and a plane connecting three key vertebrae (apex and end vertebrae) projected onto the transverse plane (Aubin, Lenke et al., 2025) (Fig. 1b). ORPD describes the spatial orientation of each curve (PT, MT, TL/L) considered as a discrete region. This captures both coronal and sagittal components of the deformity. Angles are reported as positive in the usual AIS directions for a typical right thoracic–left lumbar spine: clockwise for PT, counterclockwise for MT and TL/L. The 3D Distance from Apex to End Vertebrae Line (DAEVL) corresponds to the shortest perpendicular distance from the apex to the line connecting the end vertebrae, complementing ORPD by quantifying the magnitude of apex deviation and regional convexity (Aubin, Lenke et al., 2025).

The transverse plane local modifier is based on the Apical Vertebral Rotation (AVR), which measures axial rotation of the apical vertebra for each of the PT, MT, and TL/L spine region (Aubin, Lenke et al., 2025) (Fig. 1c). AVR is a measure of a single vertebra rotated in space, defined as the angle between the vertebral posterior–anterior axis and the global sagittal plane (i.e. plane perpendicular to the line joining both hips, to ensure independence from patient positioning), as derived from 3D reconstructions. Positive values indicate clockwise rotation for MT and counterclockwise for PT and TL/L (viewed from above), in accordance with standard scoliosis conventions [4].

These descriptors are compatible with standard radiographic workflows and 3D reconstruction methods [25, 26], facilitating clinical integration. Based on measured values, ORPD and AVR are converted into discrete 3-tier modifiers (Aubin, Lenke et al., 2025). ORPD subclasses are: mild (1, < 70°), moderate (2; 70–90°), and severe (3; ≥  90°) (Fig. 1b). Values < 70° generally indicate preserved sagittal alignment, 70–90° a transition zone often linked to sagittal flattening, and  ≥ 90° a curvature inversion relative to the regional coronal plane. AVR is stratified as slight (s; < 10°), moderate (m; 10–20°), and large (l;  ≥ 20°) (Fig. 1c). Graphical representations (Fig. 1) support interpretation and consistency. The design emphasizes simplicity, clinical interpretability, and complementarity with the original 2D Lenke framework (Aubin, Lenke et al., 2025).

### Statistical analyses of independence and complementarity

Comprehensive statistical analyses were carried out to determine whether the newly introduced SRS-Lenke-Aubin 3D descriptors offer independent, non-redundant information that complements the existing Lenke classification and enhances the multidimensional assessment of AIS.

A retrospective cohort of 285 surgical AIS patients was analyzed (Table 1). All patients had previously undergone biplanar imaging, and data usage was approved by the institutional review board (IRB) of the lead institution. Imaging data had been acquired in clinical or research contexts with appropriate informed consent.

**Table 1:** Clinical and demographic characteristics of the 285 cases (mean; min–max) – distribution of cases according to Lenke classification

	Number of cases (percentage)	Cobb angles			TK	LL	ORPD			AVR		
		PT	MT	TL/L			PT	MT	TL/L	PT	MT	TL/L
Lenke 1	101 (35.4%)	25.8 (4–49)	56.0 (34–87)	32.2 (17–61)	16.8 (-29–68)	46.0 (19–81)	68.9 (27–113)	79.5 (50–102)	57.6 (13–99)	5.0 (-15–25)	15.2 (0–36)	6.5 (-8–28)
Lenke 2	60 (21.1%)	34.5 (15–58)	65.3 (39–88)	34.5 (20–53)	13.9 (-10–51)	45.2 (18–70)	73.6 (29–101)	79.3 (55–100)	54.9 (12–105)	5.8 (-13–21)	17.2 (2–31)	4.5 (-18–17)
Lenke 3	40 (14.0%)	26.8 (8–38)	65.0 (47–82)	41.6 (18–72)	20.6 (-8–52)	51.1 (19–89)	74.7 (12–133)	78.4 (60–106)	70.3 (26–113)	4.0 (-13–14)	16.8 (1–33)	9.2 (-4–27)
Lenke 4	22 (7.7%)	31.9 (5–51)	68.8 (29–90)	48.8 (31–89)	21.4 (0–47)	51.2 (23–79)	69.8 (33–97)	76.2 (59–99)	71.5 (38–113)	3.4 (-18–29)	11.7 (-29–32)	12.0 (1–35)
Lenke 5	37 (13.0%)	8.7 (1–22)	27.8 (4–57)	45.0 (25–67)	24.0 (0–50)	43.8 (25–67)	73.5 (7–160)	60.5 (11–93)	95.9 (72–116)	3.3 (-11–16)	1.7 (-10–14)	19.0 (5–31)
Lenke 6	25 (8.8%)	12.9 (2–28)	47.4 (33–72)	62.7 (40–90)	28.0 (-8–65)	45.1 (15–63)	75.1 (22–131)	76.9 (45–45)	100.5 (83–117)	5.8 (-2–19)	6.1 (-6–18)	20.1 (-32–36)
**ALL**	**285 (100%)**	**24.9 (1–58)**	**55.8 (4–90)**	**39.6 (17–90)**	**19.0 (-29–68)**	**46.6 (15–89)**	**71.9 (7–160)**	**76.4 (11–106)**	**68.6 (12–117)**	**4.7 (-18–29)**	**13.0 (-29–36)**	**9.7 (-32–36)**

The anatomical landmarks required for 2D and 3D measurements were digitized using validated in-house software [16], and 3D reconstructions were performed using self-calibrated algorithms applied to the biplanar radiographs [25]. These digitization and reconstruction methods have been extensively validated, with previous studies reporting angular accuracy typically within 2–5° for most spinal measures [25], [27, 28], which is well within clinically accepted variability. For each spinal region—PT, MT, and TL/L—the above-described transverse plane descriptors (ORPD, AVR, and DAEVL) and their subclass modifiers (ORPD 1–3; AVR s, m, l) were computed. The distribution of these descriptors and modifiers was examined across the cohort, and cross-tabulations were used to evaluate their relationship with the original Lenke lumbar and sagittal modifiers.

To explore the independence and potential complementarity of the new 3D descriptors, a series of targeted statistical analyses were performed using RStudio (version 4.3.2, RStudio, Boston, MA). All datasets were included in the analysis. All variables were normally distributed, while skewness (asymmetry of the distribution) and kurtosis (tailedness/peakedness of the distribution) indices were all ± 2 and ± 7, respectively [20]. To minimize the influence of extreme outliers, a winsorizing method was applied: all extreme scores exceeding ± 3.29 standard deviations were identified and replaced with values capped at the ± 3.29 threshold (z =  ± 3.29), thereby minimizing the influence of outliers while retaining the structure of the dataset [21].

All primary analyses were based on Pearson correlations. Coefficients (r) were used to quantify the strength and direction of association between variables. Interpretative thresholds followed Cohen’s guidelines: r < 0.1 (negligible), 0.1–0.3 (weak), 0.3–0.5 (moderate), 0.5–0.7 (moderate to elevated), and > 0.7 (strong) [19]. Statistical significance was set at p < 0.05. Original p-values are reported. To account for multiple comparisons, correlations that reached statistical significance were subsequently verified against Holm–Bonferroni adjusted thresholds, which offer greater statistical power than the standard Bonferroni correction [17, 18].

The first analysis compared ORPD and AVR within each spinal region (PT, MT, TL/L) to evaluate whether regional plane orientation is associated with the magnitude of AVR, based on the hypothesis that greater spatial deviation might correlate with increased axial torsion. The second set of correlations examined the relationship between Cobb angle and both ORPD and AVR to assess whether traditional coronal plane metrics capture underlying transverse plane regional and local deformities. Third, the influence of sagittal alignment on transverse plane metrics was investigated by testing correlations between thoracic kyphosis (TK) and ORPD in the MT region, and between lumbar lordosis (LL) and ORPD in the TL/L region. The fourth analysis explored the spatial parameter DAEVL in relation to both Cobb angle and ORPD, aiming to provide additional insight into 3D curve morphometry.

To assess the relationship between traditional 2D Lenke modifiers (lumbar spine and thoracic sagittal profile) and their corresponding 3D transverse plane counterparts (ORPD in TL/L and MT regions), contingency tables were constructed to compare the distribution of each pair of categorical variables. A chi-square (χ2) test of independence— a non-parametric method suitable for categorical data—was then applied to determine whether a statistically significant association existed between the 2D Lenke and the 3D-based (transverse plane) modifiers. To further quantify the strength of association, Cramér’s V was calculated—a normalized measure that ranges from 0 (no association) to 1 (perfect association). This analytical approach allowed for the assessment of whether the 3D ORPD-based modifiers captured unique versus overlapping information relative to Lenke’s original lumbar and thoracic sagittal profile modifiers.

Given the dual geometric nature of ORPD—encompassing both coronal (medial–lateral) deviation and sagittal curvature—multiple linear regression models were constructed to evaluate the independent contribution of each component. For the MT region, Cobb angle and TK were included as predictors of MT’s ORPD. In the TL/L region, Cobb angle and LL were tested as independent variables predicting TL/L’s ORPD. To ensure the absence of problematic multicollinearity, we confirmed that pairwise correlations remained below 0.8, variance inflation factors (VIF) were under 5, and the conditioning index did not exceed 30 [22–24]. These thresholds ensured the robustness of the regression estimates and minimized the risk of inflated standard errors due to collinearity among predictors.

## Results

**Fig. 2:**
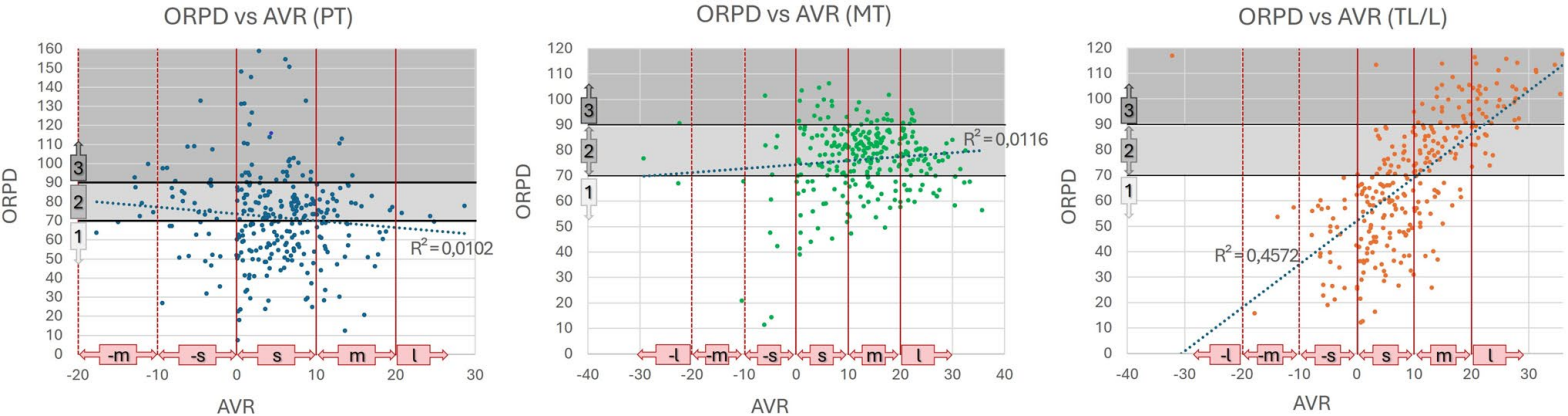
Scatter plots showing the distribution of transverse plane deformities in the PT, MT, and TL/L regions. Each point represents a patient case, plotted according to ORPD and AVR subclass combinations. The full 3 × 3 matrix (1 s to 3 l) is represented for MT, with nearly all combinations also observed for PT and TL/L. Vertical (red) and horizontal (black) lines denote the ORPD (70°, 90°) and AVR (10°, 20°) thresholds used to define subclasses 1–3 and s–m–l, respectively, including cases presenting negative (inverse) AVR values

Analyses of the 285-case AIS surgical cohort confirmed the broad variability of transverse plane deformities across the PT, MT, and TL/L spinal regions. For the MT region, all nine possible subclass combinations of the transverse plane matrix (1 s to 3 l) were identified, while nearly all combinations (8 of 9) were also observed for both PT and TL/L, underscoring the system’s capacity to capture a wide spectrum of deformity patterns without prior stratification by Lenke type (Fig. 2).

**Fig. 3:**
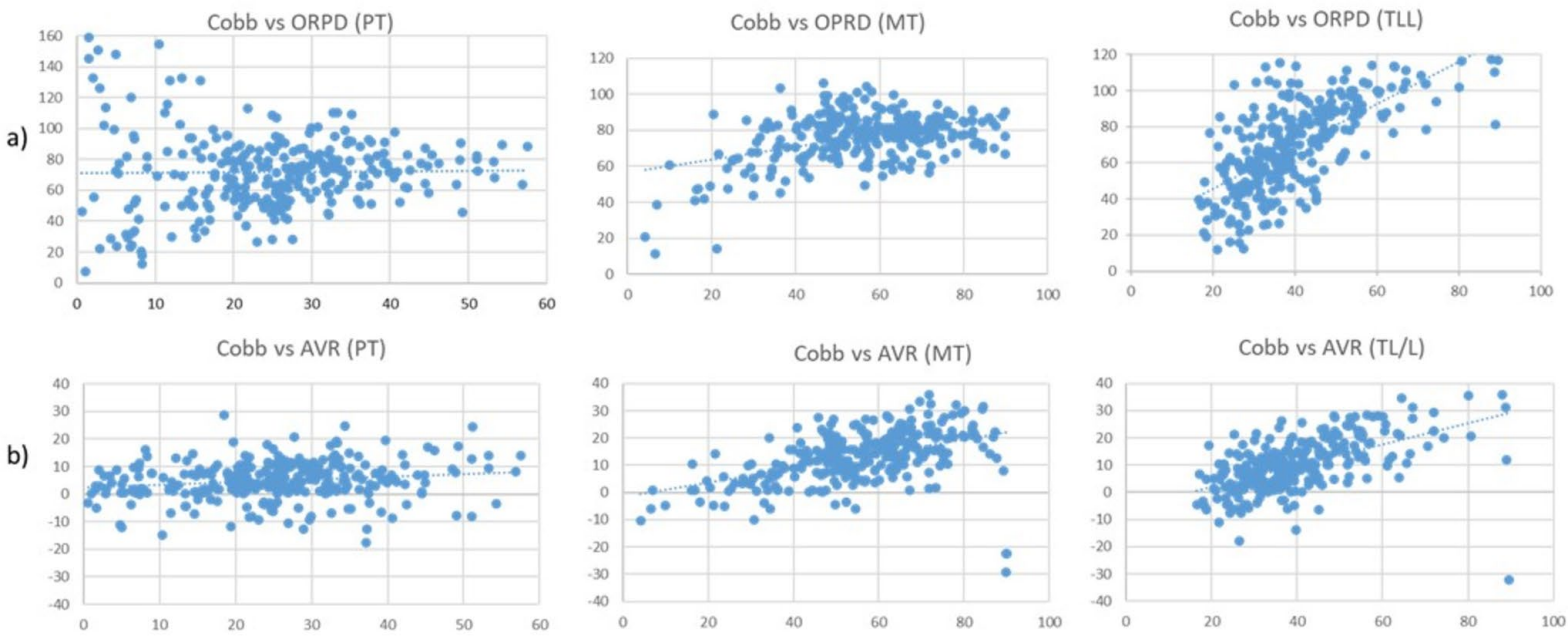
Correlation graphs showing the relationships between Cobb angles (horizontal axis) and transverse plane indices (ORPD and AVR, vertical axis) across PT, MT, and TL/L regions, with Panel a (top row) depicting Cobb vs. ORPD and Panel b (bottom row) depicting Cobb vs. AVR

ORPD values ranged from well below 70° (ORPD 1), often corresponding to a predominant sagittal component (although this was not systematically observed), to greater than 90° (ORPD 3), reflecting pronounced lateral deviation and inversion of the regional sagittal curvature (Figs. 2 and 3). AVR values ranged from below 10° (s) to beyond 20° (l), including negative values, illustrating the heterogeneity of local vertebral torsion across patients (Table 1). Notably, within a given ORPD category, patients exhibited a wide range of AVR values, further reinforcing the independence and complementarity of these two transverse plane metrics (Fig. 2).

Correlation analyses were conducted to evaluate the dimensional independence and complementarity of the new 3D descriptors relative to traditional 2D parameters following the targeted methodological framework above described, and the complete set of region-specific correlation coefficients and p-values is consolidated in Table 2.

**Table 2:** Region-specific correlation matrix reporting Pearson correlation coefficients (r), with original p-values shown in parentheses on the line below; correlations originally significant were subsequently verified and remained significant under Holm–Bonferroni adjusted thresholds for multiple comparisons.

	ORPD (PT, MT or TL/L)	AVR (PT, MT or TL/L)	DAEVL (PT, MT or TL/L)	TK	LL
Cobb PT	0.0159 (0.7887)	0.1653 (0.0052)	0.6857 (< 0.001)	−0.0576 (0.3327)	−
Cobb MT	0.4311 (< 0.001)	0.4969 (< 0.001)	0.6347 (< 0.001)	−0.1970 (< 0.001)	
Cobb TL/L	0.6673 (< 0.001)	0.5888 (< 0.001)	0.5315 (< 0.001)		−0.0656 (0.2694)
ORPD PT		−0.1000 (0.0912)	−0.1173 (0.0478)	0.0253 (0.6703)	
ORPD MT		0.0891 (0.1335)	0.3635 (< 0.001)	−0.5809 (< 0.001)	
ORPD TL/L		0.6936 (< 0.001)	0.2999 (< 0.001)		−0.0128 (0.8293)
AVR PT	−0.1000 (0.0912)		0.1480 (0.0124)	−0.0134 (0.8220)	
AVR MT	0.0891 (0.1335)		0.2809 (< 0.001)	−0.1244 (0.0358)	
AVR TL/L	0.6936 (< 0.001)		0.3574 (< 0.001)		0.0928 (0.1180)
DAEVL PT	−0.1173 (0.0478)	0.1480 (0.0124)		0.0256 (0.6665)	
DAEVL MT	0.3635 (< 0.001)	0.2809 (< 0.001)		−0.3465 (< 0.001)	
DAEVL TL/L	0.2999 (< 0.001)	0.3574 (< 0.001)			0.2204 (< 0.001)

For each column (ORPD, AVR, DAEVL), the table provides correlations with the region-appropriate 2D parameters listed in the rows for the PT, MT, and TL/L regions, consistent with the anatomical logic of the SRS-Lenke-Aubin 3D classification

The relationship between ORPD and AVR was first examined across spinal regions. Pearson correlation analyses revealed no meaningful association in the PT and MT regions (r < 0.1; p > 0.05), whereas the TL/L region demonstrated an elevated and statistically significant correlation (r = 0.6936; p < 0.01), indicating a region-specific relationship restricted to the TL/L curve (Fig. 2; Table 2).

Pearson correlations between Cobb angle and ORPD revealed no association for the PT region (r = 0.0159; p > 0.05), a moderate correlation for the MT region (r = 0.4311; p < 0.01), and an elevated correlation for the TL/L region (r = 0.6673; p < 0.01) (Fig. 3a; Table 2). Evaluation of the relationship between Cobb angle and AVR revealed weak correlations for the PT region (r = 0.1653; p < 0.01), and moderate for the MT region (r = 0.4969; p < 0.01) and for the TL/L region (r = 0.5888; p < 0.01); all correlations were statistically significant (Fig. 3b; Table 2).

**Fig. 4:**
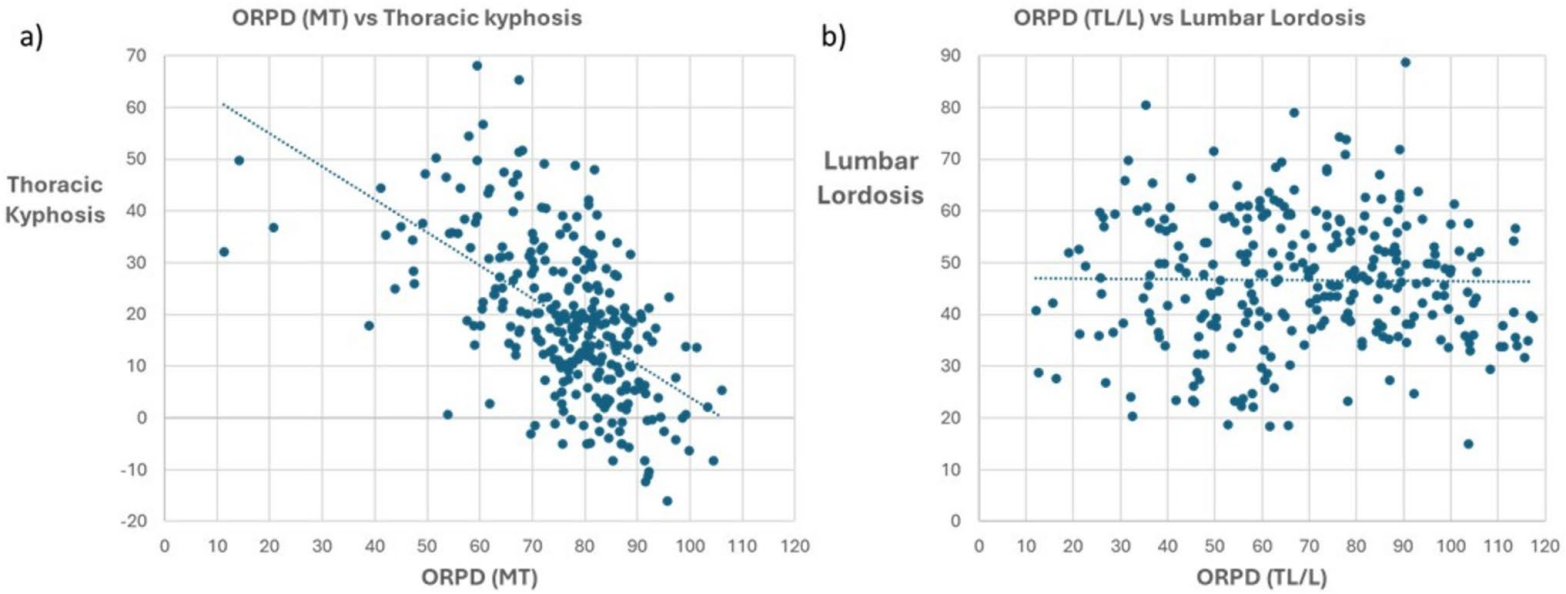
Correlation graphs illustrating the relationships between ORPD and sagittal parameters: **a** ORPD in MT region vs. TK; **b** ORPD in the TL/L spine region vs. LL

The influence of sagittal curvatures on transverse plane metrics was subsequently assessed. A moderate and statistically significant negative correlation was observed between TK and ORPD in the MT region (r = -0.5809; p < 0.01) (Fig. 4a; Table 2), suggesting that reduced kyphosis is associated with increased regional transverse deviation in the thoracic spine. In contrast, no correlation was found between LL and ORPD in the TL/L region (r = -0.0128; p > 0.05) (Fig. 4b; Table 2), indicating no meaningful relationship in the lumbar region.

**Fig. 5:**
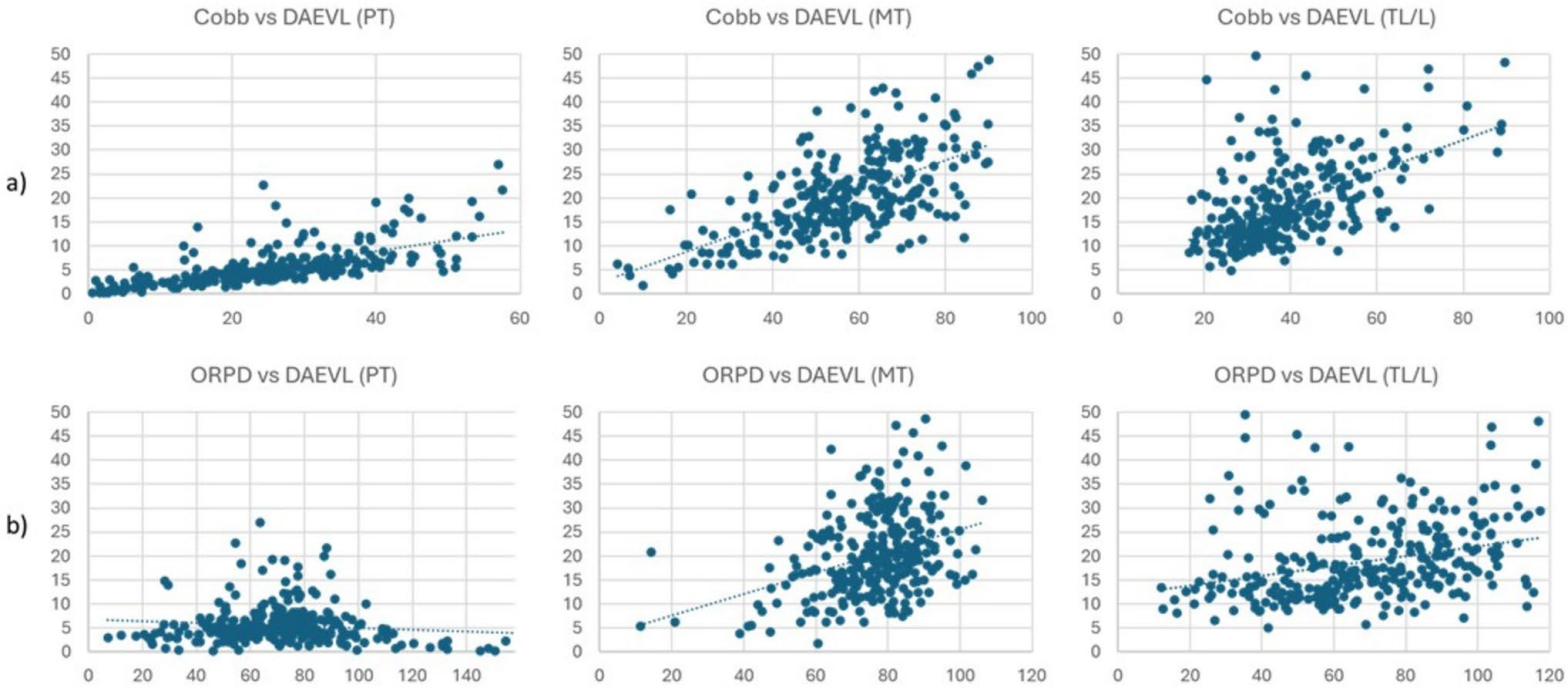
Scatter plots illustrating the relationships between **a** Cobb angle (horizontal axis) and DAEVL (vertical axis), and **b** ORPD (horizontal axis) and DAEVL (vertical axis)) across PT, MT, and TL/L regions. Panel **a** shows moderate to elevated correlations, indicating that greater coronal deformity is generally associated with increased apical displacement in the RPD. In contrast, panel **b** shows weak or moderate correlations between ORPD and DAEVL, reflecting limited spatial alignment between regional plane orientation and apex displacement within the 3D-oriented RPD

The spatial relationship between DAEVL and both Cobb angle and ORPD was also assessed. Correlations between Cobb angle and DAEVL (Fig. 5a; Table 2) ranged from moderate to elevated across all regions—PT (r = 0.6857; p < 0.01), MT (r = 0.6347; p < 0.01), and TL/L (r = 0.5315; p < 0.01)—indicating that larger coronal angular deformities are generally associated with greater apical displacement from the end-vertebrae line. In contrast, correlations between ORPD and DAEVL (Fig. 5b; Table 2) were weak in the PT (r = -0.1173, p < 0.05) and TL/L (r = 0.2999, p < 0.01) regions, while a moderate but significant correlation was observed in the MT region (r = 0.3635, p < 0.01). These findings suggest limited spatial coupling between ORPD and apex deviation along the end-vertebrae line in 3D space.

The chi-square tests and Cramér’s V analyses confirmed highly significant associations between Lenke’s traditional modifiers and their respective ORPD counterparts. A strong association was observed between Lenke’s lumbar modifier and the TL/L ORPD modifier (χ2 = 199.1, df = 4, p < 0.001; Cramér’s V = 0.59), whereas a moderate association was found between Lenke’s thoracic sagittal profile modifier and the MT ORPD modifier (χ2 = 79.1, df = 4, p < 0.001; Cramér’s V = 0.37). In the lumbar group, consistent correspondence was noted for A and B types, while marked discrepancies appeared for C curves (e.g., observed 4 vs. expected 57 in ORPD1, and observed 57 vs. expected 24 in ORPD3). In the thoracic group, overall alignment was good, but notable deviations were found in certain thoracic sagittal profile/ORPD modifier combinations (e.g., observed 23 vs. expected 9 for hypokyphosis with ORPD3). These patterns underscore both the complementarity and the added discriminative value of transverse plane descriptors relative to the Lenke framework.

Multivariate linear regression analyses confirmed that both coronal and sagittal parameters independently contribute to the ORPD, with region-specific differences in explanatory power. For the MT region, the combined model including Cobb angle and TK yielded an adjusted R2 of 0.4377 (F(2,282) = 111.5, p < 0.01), indicating that approximately 44% of the variance in ORPD of the MT region was explained by these two factors. Within this model, both Cobb MT (β = 0.25; p < 0.01) and TK (β = -0.44; p < 0.01) were significantly and independently associated with ORPD, with opposite directional effects. In the TL/L region, the model including the Cobb angle and LL yielded a comparable adjusted R^2^ of 0.4423 F(2, 282) = 113.6, p < 0.01). However, in this case, only Cobb TL/L (β = 1.20; p < 0.01) was significantly associated with the ORPD (TL/L), while LL was not (> 0.05). These findings suggest that the contribution of sagittal parameters varies by region, and that transverse plane deformity in the TL/L region is predominantly influenced by the coronal plane.

## Discussion

The new SRS-Lenke-Aubin 3D classification (Aubin, Lenke et al., 2025) represents an evolution of the established Lenke system [1, 2], integrating disruptive descriptors of the transverse plane that capture key aspects of scoliosis deformity. Specifically, it characterizes regional deviations through ORPD and local spinal torsion through AVR, thereby addressing distinct yet complementary dimensions of the three-dimensional complexity of AIS. While the original Lenke classification provides a reliable 2D modular approach based on coronal and sagittal parameters, it does not explicitly address transverse plane components, which are biomechanically and surgically relevant. In keeping with the design principles defined by the SRS Task Force, the 3D extension was therefore built to complement rather than replace the Lenke structure: curve type and structurality continue to be determined strictly according to the conventional Lenke methodology, while the transverse plane descriptors (ORPD and AVR) are applied afterward as additional 3D information. This approach preserves the familiar clinical logic of Lenke while adding optional 3D detail that supports both clinical interpretation and research applications. However, because regions are defined by the coronal end vertebrae, the corresponding sagittal region may differ from the conventional vertebral ranges used to define TK (T5–T12) or LL (L1–L5), which may explain some less intuitive ORPD modifiers when compared with the interpretation of traditional sagittal curves.

By introducing ORPD and AVR as standardized indices—which serve as additional modifiers within the extended classification system—this framework enriches the three-dimensional descriptive resolution of AIS, in line with earlier perspectives emphasizing the clinical importance of transverse plane metrics in scoliosis management [4]. An additional index, DAEVL, was also introduced. Although not retained as a formal modifier, DAEVL plays a distinct role as a descriptive—but not classificatory—3D index, quantifying apex displacement within the RPD and functioning as a spatial analogue of the apical vertebra translation (AVT), it was therefore intentionally kept outside the core modifier set given its strong functional overlap with curve-magnitude measures such as Cobb angle. Future studies could investigate DAEVL ratios—particularly values above or below 1.2—which may be more relevant than AVT ratios, an index in the coronal plane, for guiding selective fusion strategies and anticipating postoperative 3D decompensation. Together, these indices offer complementary perspectives on regional and local aspects of spinal deformity, supporting a more detailed and multidimensional interpretation of the scoliotic spine.

A key feature of this new framework is the distinction between regional deformity (ORPD) and local vertebral rotation (AVR), each assessed independently in the PT, MT, and TL/L regions. In simple terms, ORPD characterizes the spatial orientation of an entire curve, whereas AVR quantifies the rotation of the individual apical vertebra. Our findings confirm that these indices capture largely complementary aspects of 3D spinal deformity. Although several correlations were weak, many were of moderate to occasionally high magnitude (notably in the TL/L curve). Overall, this pattern suggests that ORPD and AVR tend to capture distinct, though partially overlapping, clinically synergistic features of spinal deformity, offering complementary insights into its structural and rotational characteristics. The proposed transverse-plane modifiers were intentionally selected as a minimal yet clinically interpretable set, independent of patient position, recognizing that additional 3D features of spinal deformity exist and warrant investigation in future studies. This complementary interplay enables a more nuanced characterization of deformity patterns, in which regional misalignment and local vertebral rotation may diverge and provide distinct clinical insights. The weak to moderate correlations observed in the PT and MT regions further support the conceptual distinction of these two descriptors and justify their concurrent use within a comprehensive classification system.

In the TL/L region, the observed elevated correlation is conceptually coherent given the relatively simple and lordotic morphology of the lumbar spine, where regional deviation and local rotation may often co-occur; however, substantial case-level divergence remains clinically meaningful, as illustrated in Fig. 2c, with several TL/L cases showing similar ORPD values but markedly different AVR subclasses, and conversely, curves with comparable AVR magnitudes presenting distinct regional orientations. Across all three regions (Fig. 2), this variability underscores that ORPD and AVR do not scale uniformly and therefore must be assessed together to capture the full structural and torsional complexity of AIS.

Stronger correlations were observed between DAEVL and Cobb angle, as both metrics quantify the regional inflection of the scoliotic curve—one in the coronal plane, the other in the 3D RPD. While Cobb angle within the RPD could potentially serve as an alternative index, DAEVL appears to better reflect the spatial configuration relevant to surgical planning, particularly in identifying the apex displacement to be addressed.

Building on the results of the statistical analyses comparing Lenke’s lumbar spine and thoracic sagittal profile modifiers with their respective ORPD counterparts, the findings confirm that the newly proposed transverse plane ORPD modifiers are statistically associated with their traditional 2D counterparts, which is logically expected given that the lumbar spine modifier partly reflects the spatial lateral displacement described by the ORPD, yet they convey distinct information. The strong association in the lumbar region (Cramér’s V = 0.59) and the moderate association in the thoracic region (Cramér’s V = 0.37) suggest that the ORPD modifiers provide complementary spatial insights. Incorporating these transverse plane characteristics—ideally in conjunction with the corresponding quantitative angular values—may enable a more comprehensive 3D characterization of scoliosis. Retaining both sets of modifiers, along with numerical angle data, could therefore improve clinical interpretation and support more tailored surgical strategies for individual deformity patterns.

Multivariate regression analyses further support the value of combining coronal and sagittal inputs to explain transverse plane deformity, while revealing important region-specific differences. In the MT region, Cobb angle and TK together explained a substantial portion of ORPD variability, with opposite directional effects, whereas in the TL/L region, ORPD variability was driven predominantly by coronal magnitude. Importantly, in both regions, more than half of the variance in ORPD remained unexplained by conventional 2D parameters, underscoring that ORPD conveys complementary 3D information not reducible to traditional coronal or sagittal metrics. In the MT region, the opposite associations with TK (β = –0.44) and Cobb angle (β = 0.25) further suggest that ORPD reflects a complex spatial interplay between planes that cannot be inferred from isolated 2D measurements. This residual variability likely reflects additional biomechanical and anatomical influences not captured by standard radiographic measures, including regional differences in torsional stiffness, 3D vertebral and disc wedging asymmetry, rib cage morphology in the thoracic spine, pelvic and lumbosacral alignment in the lumbar region, and growth-related torsional forces implicated in AIS progression. These mechanisms should be regarded as plausible but speculative contributors rather than proven determinants; however, their conceptual consistency with prior biomechanical observations in AIS underscores why dedicated transverse-plane descriptors such as ORPD and AVR are required to capture aspects of 3D deformity not reflected in conventional coronal and sagittal metrics.

Rather than duplicating existing metrics, ORPD emerges as a truly 3D descriptor of transverse morphology that complements traditional parameters. The model confirms that while coronal and sagittal inputs are statistically associated with ORPD, they do not fully account for its dimensional content. This finding supports the rationale for maintaining ORPD as a distinct and complementary index within the proposed SRS-Lenke-Aubin 3D classification system. By extension, ORPD contributes to a more comprehensive characterization of AIS, enabling a refined interpretation of bi-planar regional deviations that may enhance patient-specific planning and surgical decision-making—especially in cases where 2D descriptors fail to capture the full geometric complexity of the deformity.

Furthermore, integrating discrete ORPD and AVR modifiers extends the modularity of the Lenke system while preserving its clinical coherence. This pragmatic design addresses limitations of previous 3D classifications—such as excessive complexity and poor interpretability—by providing complementary transverse plane information alongside traditional sagittal and lumbar modifiers. This combination enhances the system’s ability to describe structural complexity across all three planes, offering a more refined framework for interpretation and surgical planning.

Operationally, it retains the simplicity and logic of the Lenke framework while adding only two transverse plane descriptors, each stratified into three levels, balancing usability with descriptive depth. The presence of nearly all ORPD/AVR combinations in the cohort highlights its capacity to distinguish diverse deformity patterns, underscoring its practical utility for capturing clinically meaningful variation in AIS.

Although the present study focuses on surgically treated AIS cases—consistent with the intended clinical target of the SRS-Lenke-Aubin 3D classification—the geometric descriptors themselves are not surgery-specific and capture fundamental transverse-plane features that may also be relevant in conservatively managed patients; evaluating their behavior in non-operative populations therefore represents an important direction for future work. Future studies of the SRS 3D Classification Task Force will also explore how the classification may be applied across evolving surgical philosophies and emerging techniques (e.g., Vertebral Body Tethering or Anterior Scoliosis Correction), thereby further extending its clinical relevance beyond the scope of the present analysis.

**Fig. 6:**
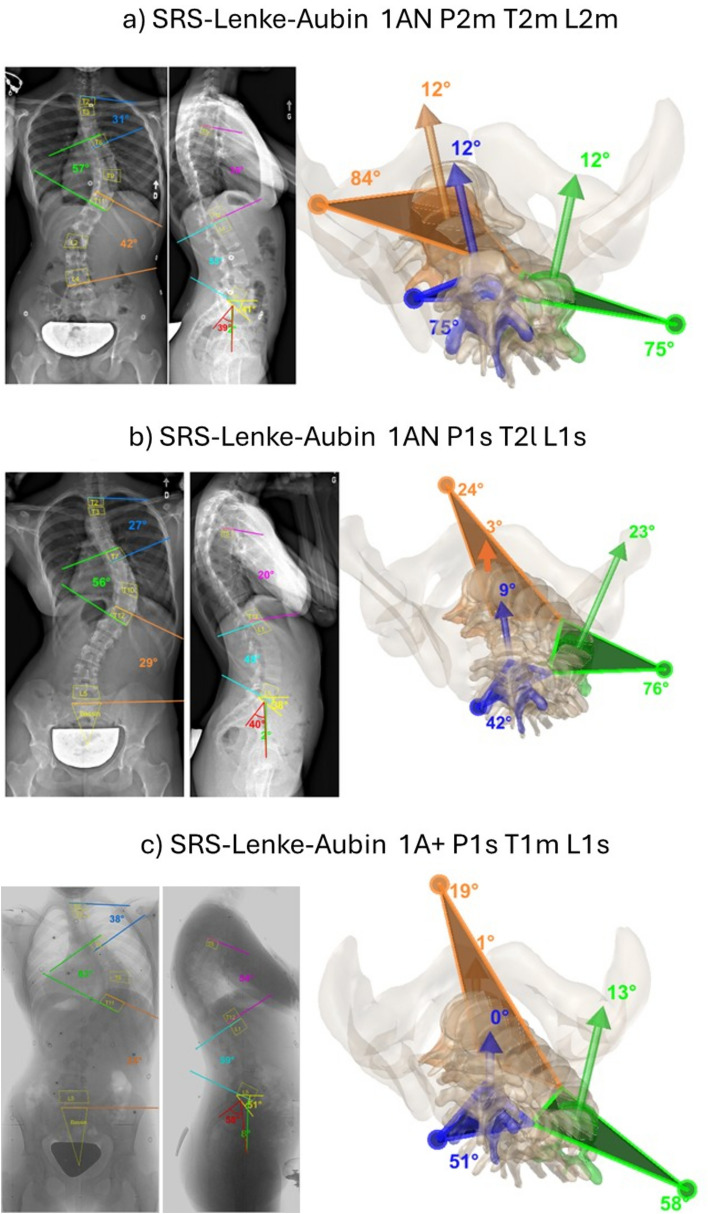
Examples of Lenke 1A cases with comparable coronal Cobb magnitudes but distinct transverse-plane morphologies. Panel **a** illustrates a configuration in which both TL/L and MT ORPD are pronounced, and associated with moderate AVR (T2m L2m), reflecting greater global malalignment. This case might therefore require a more extensive fusion to correct the TL/L curve. In contrast, panels (**b**) and (**c**) show cases with lower TL/L ORPD and AVR (L1s), suggesting that some similar coronal curves may instead be candidates for shorter selective fusions. The differing degrees of MT ORPD and AVR (T2l vs. T1m) might also be used by surgeons to select the optimal curve reduction technique (derotation in T2l or posteromedial translation in T1m)

Clinically, this multidimensional profiling supports tailored surgical strategies. As further illustrated in Fig. 6, Lenke 1A cases with comparable coronal Cobb magnitudes but distinct ORPD–AVR combinations demonstrate how transverse-plane morphology may influence surgical considerations beyond 2D classification alone. In panel (a), both TL/L and MT ORPD are similarly affected and associated with moderate AVR (T2m L2m), reflecting greater regional malalignment, and that configuration may warrant a more extensive fusion to correct the distal curve which is sagittally flattened (T2 L2). In contrast, panels (b) and (c) illustrate cases with lower TL/L ORPD and AVR (L1s), suggesting that some similar coronal curves may instead be candidates for shorter selective fusions. The differing degrees of MT ORPD and AVR (T2l vs. T1m) might also help surgeons determine the optimal corrective strategy for the MT curve (segmental derotation in case b and posteromedial translation for the less rotated case c). Together, these examples highlight how discordant ORPD–AVR patterns can inform the relative prioritization of translational versus rotational correction maneuvers within the same Lenke subtype. However, the primary role of the classification at this stage is not to prescribe treatment algorithms, but rather to provide a precise 3D morphological language and descriptors upon which future outcome-based and strategy-oriented studies can be built. Accordingly, as a next step within the SRS 3D Classification Task Force mandate, future studies will define surgery-informed strategies based on transverse-plane descriptors and modifiers, an effort that extends beyond the analytical scope of the present study.

Although the system relies on a limited number of discrete modifiers, this simplification is intentional. By categorizing continuous measures into defined thresholds, some nuance near boundary values may be lost. However, this trade-off favors clarity and clinical usability over granularity. As with the original Lenke system, where Cobb angle values still guide curve interpretation, the actual numerical values of ORPD and AVR remain relevant when applying the classification. Future enhancements may explore threshold refinement or integrate probabilistic subclassification to improve transitional zone sensitivity.

This study represents an important first step in validating the SRS-Lenke-Aubin 3D classification, but it analyzes all Lenke curve types collectively in this initial phase. In future work, it will be important to examine ORPD and AVR distributions and their relationships with other 2D indices within each Lenke class, to identify specific criteria through which transverse modifiers could add value. This study also contributes to the broader validation initiative led by the SRS 3D Classification Task Force. Parallel studies are underway to evaluate reliability, usability, and surgeon confidence—topics beyond the scope of the present report. Together, these efforts aim to establish a robust evidence base to support clinical adoption. While our findings confirm the system’s descriptive strength, further research is needed to evaluate its impact on shared clinical understanding, treatment planning, and decision-making. Ultimately, broader adoption of the classification could enhance surgical precision, improve intraoperative execution, and enable structured long-term follow-up grounded in individualized 3D deformity profiles.

## Conclusion

In summary, the new SRS-Lenke-Aubin 3D classification introduces clinically meaningful transverse plane descriptors that complement the established 2D Lenke framework. The addition of ORPD and AVR enables a more nuanced, region-specific assessment of both regional deviations and local vertebral rotation, thereby improving the dimensional accuracy of scoliosis characterization. These analyses showed negligible to moderate correlations between ORPD and AVR in most regions, with a moderate to elevated correlation in TL/L, and region-specific relationships with coronal and sagittal parameters. Moderate to elevated correlations between Cobb angle and DAEVL suggest that DAEVL may have particular relevance for surgical planning. Rather than duplicating existing modifiers, these descriptors provide synergistic information. They form a foundational tier of a broader, expandable 3D classification structure that may ultimately support a transition toward more comprehensive, individualized, and biomechanically informed management of AIS, a potential impact that will need to be confirmed in future outcome-based studies. This first step lays the groundwork for a scalable framework that could, in future iterations, integrate additional descriptors—such as advanced and more nuanced 3D indices, along with pelvic and thoracic cage parameters—to further refine the clinical understanding and treatment of AIS across all three anatomical planes.

## Data Availability

In the spirit of open science, inquiries regarding potential use of the data for future research may be directed to the corresponding author. Access is subject to ongoing studies of the SRS 3D Classification Task Force currently making use of these datasets.

## References

[1] Lenke LG, Betz RR, Harms J et al (2001) Adolescent idiopathic scoliosis: a new classification to determine extent of spinal arthrodesis. J Bone Joint Surg Am 83(8):1169–1181 11507125

[2] Lenke LG, Edwards CC, Bridwell KH (2003) The Lenke classification of adolescent idiopathic scoliosis: how it organizes curve patterns as a template to perform selective fusions of the spine. Spine (Phila Pa 1976) 28(20):199–207 10.1097/01.BRS.0000092216.16155.33 14560193

[3] Lenke LG, Lee V, Hassan FM (2024) Revision of surgery for adolescent idiopathic scoliosis: reasons, treatments, and clinical management with case examples. J Clin Med 13(8):2233 38673506 10.3390/jcm13082233 PMC11051103

[4] Labelle H, Aubin CE, Parent S et al (2011) Seeing the spine in 3D: how will it change what we do? J Pediatr Orthop 31(1 Suppl):S37–S45 21173617 10.1097/BPO.0b013e3181fd8801

[5] Stokes IA, Sangole AP, Aubin CE (2009) Classification of scoliosis deformity: three-dimensional spinal shape by cluster analysis. Spine (Phila Pa 1976) 34(6):584–590 19282737 10.1097/BRS.0b013e318190b914 PMC2664249

[6] Duong L, Cheriet F, Labelle H (2006) Three-dimensional classification of spinal deformities using fuzzy clustering. Spine (Phila Pa 1976) 31(8):923–930 16622383 10.1097/01.brs.0000209312.62384.c1

[7] Duong L, Mac-Thiong JM, Cheriet F, Labelle H (2009) Three-dimensional subclassification of Lenke type 1 scoliotic curves. J Spinal Disord Tech 22(2):135–143 19342936 10.1097/BSD.0b013e31816845bc

[8] Sangole AP, Aubin CE, Labelle H, Stokes IA, Lenke LG, Jackson R, Newton P (2009) Three-dimensional classification of thoracic scoliotic curves. Spine (Phila Pa 1976) 34(1):91–99 19127167 10.1097/BRS.0b013e3181877bbb

[9] Kadoury S, Labelle H (2012) Classification of 3D thoracic deformities in AIS from a multivariate analysis. Eur Spine J 21(1):40–49 21879413 10.1007/s00586-011-2004-2 PMC3252447

[10] Shen J, Parent S, Wu J, Aubin CÉ et al (2020) Towards a new 3D classification for adolescent idiopathic scoliosis. Spine Deform 8(3):387–396 32026444 10.1007/s43390-020-00051-2

[11] Pasha S, Flynn J (2018) Data-driven classification of the 3D spinal curve in AIS with application in surgical outcome prediction. Sci Rep 8:16296 30389972 10.1038/s41598-018-34261-6 PMC6214965

[12] Pasha S, Hassanzadeh P, Ecker M et al (2019) A hierarchical classification of AIS: identifying distinguishing features in 3D spinal deformities. PLoS ONE 14(3):e0213406 30893327 10.1371/journal.pone.0213406 PMC6426223

[13] Donzelli S, Poma S, Balzarini L et al (2015) State of the art of current 3-D scoliosis classifications: a systematic review from a clinical perspective. J Neuroeng Rehabil 12:91 26475324 10.1186/s12984-015-0083-8 PMC4609046

[14] Wei W, Cheng L, Dong Y, Zhang T, Deng Y, Gong J, Xie F, Yang J (2025) 2D and 3D classification systems for adolescent idiopathic scoliosis: clinical implications and technological advances. Orthop Surg 17(4):999–1020 39825698 10.1111/os.14362 PMC11962298

[15] Aubin CE, Lenke LG, Vitale M et al (2025) The SRS-Lenke-Aubin 3D classification of adolescent idiopathic scoliosis. Spine Deform. 10.1007/s43390-025-01253-2 41400792 10.1007/s43390-025-01253-2 PMC13282214

[16] Aubin CE, Bellefleur C, Joncas J, de Lanauze D, Kadoury S, Blanke K, Parent S, Labelle H (2011) Reliability and accuracy analysis of a new semiautomatic radiographic measurement software in adult scoliosis. Spine (Phila Pa 1976) 36(12):E780–E790 21224755 10.1097/BRS.0b013e3181f0825a

[17] Chen SY, Feng Z, Yi X (2017) A general introduction to adjustment for multiple comparisons. J Thorac Dis 9:1725–1729 28740688 10.21037/jtd.2017.05.34 PMC5506159

[18] Holm S (1979) A simple sequentially rejective multiple test procedure. Scand J Stat 6:65–70

[19] Cohen J (1988) Statistical power analysis for the behavioral sciences. Erlbaum Associates, Hillsdale

[20] Curran PJ, West SG, Finch JF (1996) The robustness of test statistics to nonnormality and specification error in confirmatory factor analysis. Psychol Methods 1:16–29

[21] Tabachnick BG, Fidell LS (2014) Using multivariate statistics. Pearson Education, Harlow

[22] Berry WD, Feldman S (1985) Multiple regression in practice. Sage Publications, Beverly Hills

[23] Belsley DA, Kuh E, Welsch RE (1980) Regression diagnostics: identifying influential data and sources of collinearity. John Wiley & Sons, New York

[24] Rogerson PA (2001) Statistical methods for geography: a student’s guide. Sage Publications, London

[25] Kadoury S, Cheriet F, Laporte C, Labelle H (2007) A versatile 3d reconstruction system of the spine and pelvis for clinical assessment of spinal deformities. Med Biol Eng Comput 45(6):591–602 17530454 10.1007/s11517-007-0182-1

[26] Cheriet F, Dansereau J, Petit Y, Aubin CÉ, Labelle H, de Guise JA (1999) Towards the self-calibration of a multiview radiographic imaging system for the 3d reconstruction of the human spine and rib cage. Int J Pattern Recognit Artif Intell 13(5):761–779

[27] Aubin CE, Lobeau D, Labelle H et al (1999) Planes of maximum deformity in the scoliotic spine. In: Stokes IAF (ed) Research into Spinal Deformities. IOS Press, Amsterdam, The Netherland, pp 45–48

[28] Boyer L, Shen J, Parent S, Kadoury S, Aubin CE (2018) Accuracy and precision of seven radiography-based measurement methods of vertebral axial rotation in adolescent idiopathic scoliosis. Spine Deform 6(4):351–357 29886904 10.1016/j.jspd.2017.12.004

